# Prospective study of the relevance of circulating tumor cell status and neoadjuvant chemotherapy effectiveness in early breast cancer

**DOI:** 10.1002/cam4.2876

**Published:** 2020-02-04

**Authors:** Chao Ni, Yimin Shen, Qingqing Fang, Min Zhang, Hongjun Yuan, Jingxia Zhang, Miaochun Zhong, Yajuan Zheng

**Affiliations:** ^1^ Department of Breast Surgery (Surgical Oncology) The Second Affiliated Hospital of Zhejiang University School of Medicine Hangzhou Zhejiang China; ^2^ Key Laboratory of Tumor Microenvironment and Immune Therapy The Second Affiliated Hospital of Zhejiang University School of Medicine Hangzhou Zhejiang China; ^3^ Department of Breast Surgery Zhejiang Provincial People's Hospital People's Hospital of Hangzhou Medical College Hangzhou Zhejiang China; ^4^ Department of Endocrinology The Second Affiliated Hospital of Zhejiang University School of Medicine Hangzhou Zhejiang China; ^5^ Department of Breast Surgery Huzhou Central Hospital Zhejiang University Huzhou Zhejiang China

**Keywords:** CanPatrol system, CTCs, early breast cancer, neoadjuvant chemotherapy

## Abstract

Although unequivocal evidence has shown the prognostic relevance of circulating tumor cells (CTCs) in patients with metastatic breast cancer (MBC), less evidence is available for its significance in neoadjuvant chemotherapy (NCT) in early breast cancer (BC). Here we conducted an analysis of individual data from 86 patients confirmed as invasive BC by core‐needle biopsy in Zhejiang Provincial People's Hospital between June 2013 and January 2017. The CTCs were assessed at the time after diagnosis and before surgery with the CanPatrol technique. The median follow‐up duration was 46.3 months. CTCs were detected in 37.2% of all patients (29/78) at baseline, and the presence of CTCs was associated with tumor size, tumor stage, and molecular classification. After NCT, the CTC‐positive patients were dropped from 29 to 8, and the EC‐T (epirubicin/cyclophosphamide followed by docetaxel) and TEC (docetaxel/epirubicin/cyclophosphamide) strategies reduce CTC‐positive patients from 16 to 3 and 13 to 5, respectively. The CTC‐negative conversion rates were similar in ER/PR+ HER2+ (5/7, 71.4%), ER/PR− HER2+ (8/11, 72.7%), and TNBC (7/10, 70%) during NCT. In addition, we explored the association between CTC‐negative conversion and objective response rate (partial response and complete response, ORR) and pathological complete response rate (pCR), and our results indicate that ORR was higher in patients with positive CTCs and converted to negative after NCT (ORR, *P* = .013; pCR, *P* = .0608). Our study preliminarily highlights the relevance of CTC status and NCT effectiveness in early BC using the CanPatrol system.

## Graphical abstract

The positive CTC result is related to patients' age, tumor size, TNM staging, and molecular classification in early breast cancer patients. The total number and CTC‐positive rate are significantly decreased after NCT in early breast cancer. CTC‐positive converted to negative after chemotherapy presents with a higher pCR rate in patients.

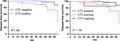

## 1 INTRODUCTION

Breast cancer (BC) is the most common cancer among women worldwide, while distant metastasis remains the major cause of mortality.^1^ Among various therapeutic strategies, neoadjuvant chemotherapy (NCT) take a core position in systematic treatment of local advanced BC.^2^ Except for its downstage effect, NCT also provides information about the chemosensitivity of tumor cells.^3^ In addition, recent reports indicate that patients who receive NCT and have residual disease could benefit from following intensive or prolonged treatment.^4^, ^5^ Multiple approaches have been applied to investigate the effectiveness of NCT, such as Ki67, MRI, and PET scanning,^6^, ^7^, ^8^, ^9^ while the potential value of CTC in NCT of BC has only been reported in a few studies.^10^

Circulating tumor cells (CTCs), which are thought to be responsible for cancer metastasis, play a pivotal role in the metastatic cascade and have been shown to be an independent prognostic factor in metastatic BC (MBC).^11^ In addition, recent studies revealed that even at very early stages, such as in situ carcinoma, which is theoretically restricted to locoregional tissue, tumor cells could invade into the circulation and begin to form metastatic lesions.^12^, ^13^ Abundant evidence proved that the persistent presence of CTCs was associated with unfavorable outcomes in BC,^14^ while CTC count ≥5/7.5 mL peripheral blood (PB) indicated an unsatisfactory treatment response.^15^ Thus, CTCs have the potential to function as a biomarker with great prognostic and predictive value for MBC,^2^ but their significance in the early stages of BC remains controversial.^16^, ^17^

The detection of CTCs has been reported with various techniques. The CellSearch System is still the only US FDA‐approved technique, and it captures CTCs by detecting epithelial cell surface markers.^18^ However, it has been reported that CTCs undergoing epithelial to mesenchymal transition could partially or completely lose epithelial markers^19^ and would exhibit greater metastatic potential, which could be omitted by the CellSearch technique.^20^ Based on our previous study with the CanPatrol system, the nonepithelial‐type CTCs could account for half of the total numbers in MBC patients.^21^ Thus, it is necessary to identify both epithelial and nonepithelial (intermediate and mesenchymal type) CTCs.

In this study, we prospectively assessed CTC numbers from 78 patients with early stage BC before and after NCT prior to surgery with the CanPatrol technique,^21^ and information on disease‐free survival (DFS) was also provided. The primary aim of this study was to evaluate whether CTCs could serve as an independent predictive factor of NCT effectiveness in early BC.

## 2 MATERIALS AND METHODS

### 2.1 Study participants

Between June 2013 and January 2017, a total of 86 female patients in Zhejiang provincial People's Hospital who were pathologically confirmed as having invasive BC by core‐needle biopsy were enrolled in this study. Eligible patients were staged at initial clinical stage (stage II‐III B), had no history of prior malignant disease, had no contraindications for chemotherapy, and consented to receive NCT and CTC assessment thereafter.

### 2.2 Study design and intervention

This study was approved by the Ethics Committee of Zhejiang Provincial People's Hospital and obtained informed consent from all patients. Patients were randomly assigned in a 1:1 ratio with the use of random number table, to receive preoperatively four cycles of epirubicin (90 mg/m^2^)/cyclophosphamide (600 mg/m^2^) followed by four cycles of docetaxel (100 mg/m^2^), or six cycles of epirubicin (75 mg/m^2^)/cyclophosphamide (600 mg/m^2^)/docetaxel (75 mg/m^2^). Patients with HER2‐positive tumors received trastuzumab (starting with a loading dose of 8 mg/kg iv and then 6 mg/kg iv every 3 weeks). The two time points of CTC assessment were before NCT and at the completion of NCT but before surgery (13‐19 days, mean ± SD = 15.06 ± 1.17 days). Pathological characteristics were obtained based on the initial core‐needle biopsy specimens before NCT. The expression of estrogen receptor (ER), progesterone receptor (PR), and human epidermal growth factor receptor 2 (HER2) was detected by immunohistochemical methods. The assessment of ER and PR receptors was in accordance with the American Society of Clinical Oncology and the American College of Pathologist guidelines.^22^ The Her2 test scoring system was used to evaluate HER2 expression.^23^ The follow‐up was made by phone call and outpatient service. In this study, overall survival (OS) was defined as the time from completion of surgery to either death or the last known date alive. DFS was defined as the time from completion of surgery to events such as relapse or metastasis, death from any cause, or the last known date alive.^24^

### 2.3 Detection of CTCs

Peripheral blood (7.5 mL) was collected from each patient in EDTA anticoagulant tubes and stored at room temperature until cell isolation, which was performed within 4 hours. CTCs were isolated by the CanPatrol CTC assay (SurExam). PB samples were treated with erythrocyte lysis buffer within 4 hours after venopuncture and filtered with an 8‐μm diameter pore calibrated membrane (EMD Millipore) to enrich the CTCs. Then, the CTCs were subjected to RNA in situ hybridization with a combination of epithelial (EpCAM and CK8/18/19) and mesenchymal (vimentin and TWIST1) markers. Finally, the samples were stained with 4′,6‐diamidino‐2‐phenylindole (Sigma‐Aldrich Co.) and analyzed with an automated imaging fluorescent microscope (Carl Zeiss Meditec AG). Then, the CTCs from each patient were classified based on the identification of the markers. Our study defined patients with ≥1 CTC per 7.5 mL of blood as CTC‐positive.

Besides, as we described before,^21^ the phenotypes of CTCs were classified into three subgroups using the RNA‐ISH method: epithelial type (E+, Cytokeratin 8,18,19+, EpCam+), mesenchymal type (M+, Vimentin+, Twist+), and biophenotypic type (B+, express both E+ and M+ markers).

### 2.4 Tumor response

Tumor response was evaluated by MRI^25^: the two greatest perpendicular diameters of the tumors in the breast and axillary nodes were measured, and the products of these diameters were added as a measure of total tumor size. No evidence of tumors in the breast and axillary lymph nodes with MRI evaluation was defined as a complete response (CR). A reduction in the sum of the products of the tumor masses of 30% or greater was classified as a clinical partial response (PR), and objective response rate (ORR) was defined as the sum of CR and PR. An increase in the sum of the products of more than 20% or the appearance of new suspicious ipsilateral axillary adenopathy was considered progressive disease. Tumors that did not meet the criteria for response or progression were classified as stable disease (SD). pCR was defined as no residual invasive cancer both in breast and axillary lymph nodes after NCT.

### 2.5 Statistical analysis

Data were analyzed using the statistical package for SPSS version 21.0 (SPSS Inc). Clinical data are expressed as percentages, and the Chi‐square test was performed for comparison within a group. All immunohistochemistry had to be centrally analyzed by FISH in one of five China reference centers, and the t‐test was used for comparisons of two groups. A significance level of *P* < .05 was set as a significant difference.

## 3 RESULTS

### 3.1 Patient characteristics and CTC evaluation at baseline

Among the 86 patients enrolled in CTC measurements before chemotherapy, five patients did not complete NCT but refer to surgery in other hospital due to insurance issue, and three patients did not complete CTC detection due to hemolysis of preoperative blood sample. Therefore, 78 patients finally completed the study. As shown in Table 1, the basic clinical characteristics were well balanced between the two groups. CTCs were detected in 37.2% of the patients (n = 29/78) at baseline (Table S1). Similar to previous reports,^26^, ^27^ the CTC‐positive detection was related to young age (*P* = .021), big tumor size (*P* < .001), and advanced grade (*P* = .002), and CTCs were more likely to be detected in TNBC and hormonal receptor‐negative subtype (*P* = .005) (Table 2). However, we did not find a significant association between menopause status, tumor site, hormone receptor, HER2 expression status, and CTCs (*P* > .05). In addition, compared with our previous data, 75% of MBC patients had CTC numbers greater than 5/7.5 mL of PB.^21^ Here, we found that 31.1% (9/29) of patients had CTC numbers greater than 5/7.5 mL of blood.

**Table 1:** The basic characteristics of breast cancer patients in two NCT strategy

Clinicopathological Characteristics		Chemotherapy EC‐>T (n = 39) (%)	Chemotherapy TEC (n = 39) (%)	χ^2^	*P*
Age (y)	≤40	6 (15.4)	8 (20.5)	0.348	.555
	>40	33 (84.6)	31 (74.5)		
Menopausal status	Premenopausal	27 (69.2)	25 (64.1)	0.231	.631
	Postmenopausal	12 (30.8)	14 (35.9)		
Primary site	Left	17 (43.6)	20 (51.3)	0.63	.427
	Right	22 (56.4)	19 (48.7)		
Tumor size	>5 CM	15 (38.5)	16 (41.0)	0.054	.817
	≤5 CM	24 (61.5)	23 (59.0)		
ER status	Positive	24 (61.5)	22 (56.4)	0.212	.645
	Negative	15 (38.5)	17 (43.6)		
PR status	Positive	22 (56.4)	20 (51.3)	0.206	.65
	Negative	17 (43.6)	19 (48.7)		
C‐erbB‐2 status	Positive	21 (53.8)	17 (43.6)	0.821	.365
	Negative	18 (46.2)	22 (56.1)		
Ki‐67expression	>20%	15 (38.5)	17 (43.6)	0.212	.645
	≤20%	24 (61.5)	22 (56.1)		
TNM staging	ⅡB	8 (10.3)	9 (11.5)	0.849	.838
	ⅢA	12 (30.8)	14 (35.9)		
	ⅢB	19 (48.7)	16 (41.0)		
CTC counts	0	22	27	−1.277	.202
	01‐May	11	9		
	05‐Oct	5	3		
	>10	1	0		
Molecular classification	Luminal A	8 (20.5)	7 (17.9)	1.498	.683
	Luminal B	10 (25.6)	15 (38.5)		
	C‐erbB 2 overexpression	12 (30.8)	10 (25.7)		
	Basal‐like	9 (23.1)	7 (17.9)		

Abbreviations: BC, breast cancer; CTC, circulating tumor cell; ER, estrogen receptor status; NCT, neoadjuvant chemotherapy; PR, progesterone receptor status; EC‐>T: four cycles of epirubicin (90 mg/m^2^)/cyclophosphamide (600 mg/m^2^) followed by four cycles of docetaxel (100mg/m^2^); TEC: six cycles of epirubicin (75 mg/m^2^)/cyclophosphamide (600 mg/m^2^)/docetaxel (75 mg/m^2^).

**Table 2:** The phenotype of CTCs before and after neoadjuvant chemotherapy

Clinicopathological Characteristics		case	CTCs‐positive (n, %)	χ^2^	*P*
Age (y)	≤40	14	9 (64.3)	5.368	.021
	>40	64	20 (31.2)		
Menopausal status	Premenopausal	52	21 (40.4)	0.346	.556
	Postmenopausal	26	8 (30.8)		
Tumor site	Left	37	12 (32.4)	0.629	.410
		41	17 (41.5)		
	Right				
Tumor size	>5 CM	31	21 (67.7)	20.575	<.001
	≤5 CM	47	8 (17.0)		
ER status	Positive	46	16 (37.8)	0.276	.599
	Negative	32	13 (40.6)		
PR status	Positive	42	15 (35.7)	0.084	.772
	Negative	36	14 (38.9)		
C‐erbB‐2 status	Positive	38	18 (47.4)	3.294	.070
	Negative	40	11 (27.5)		
Ki‐67 expression	>20%	32	12 (37.5)	0.002	.961
	≤20%	46	17 (36.9)		
Tumor grading	ⅡB	17	3 (17.6)	14.948	.002
	ⅢA	26	9 (34.6)		
	ⅢB	35	17 (48.6)		
Molecular classification	ER/PR + HER2‐	15	1	12.821	.005
	ER/PR + HER2+	25	7		
	ER/PR‐ HER2+	22	11		
	TNBC	16	10		

Abbreviations: BC, breast cancer; CTC, circulating tumor cell; ER, estrogen receptor status; HER2, human epidermal growth factor receptor 2; PR, progesterone receptor status; TNBC, triple‐negative breast cancer.

### 3.2 Dynamic changes in CTCs pre‐ and postchemotherapy

The total number of CTCs was significantly decreased after NCT (*P* < .001, Table S1), and the CTC‐positive rate dropped from 37.2% to 10.3%. Besides, our data revealed the E+ CTCs was detected in 22 patients (75.9%) before NCT, and in seven patients (23.3%) after NCT; on the other side, the non‐E+ CTCs (B+ and M+) was also detected in 22 patients (75.9%) before NCT, and in five patients (16.7%) after NCT. According to the study design, patients were grouped by two different NCT strategies (EC‐T or TEC) (Table 3). Although without a statistically significant difference, our results indicated that the EC‐T and TEC strategies reduced CTC‐positivity from 41% to 7.7% and 33.3% to 12.8%, respectively (χ^2^ = 0.58, *P* = .44). When comparing the effects of two different chemotherapy strategies, we found that the CTC‐negative conversion rate (the percentage of CTC‐positive patients changed to CTC‐negative after NCT) was similar among the two chemotherapy strategies. Furthermore, we tried to compare the CTC‐negative conversion rate between four molecular subtypes (Table 4). There was only one CTC‐positive patient in the ER/PR+ HER2‐ subtype, while the CTC‐negative conversion rate was similar among the other subtypes (ER/PR+ HER2+, 71.4% (5/7); ER/PR‐ HER2+, 72.7% (8/11); TNBC, 70% (7/10)), and one patient (ER/PR+ HER2+) was CTC‐negative at baseline and turned CTC‐positive after NCT.

**Table 3:** Comparison of CTC status pre and post two different NCT regimens

Variable		CTC‐positive (%)	CTC‐negative (%)	*P* value
Before NCT	Chemotherapy EC‐>T	16 (41)	23 (59)	.482
	Chemotherapy TEC	13 (33.3)	26 (66.7)	
	Total number	29 (37.2)	49 (62.8)	
After NCT	Chemotherapy EC‐>T	3 (7.7)	36 (92.3)	.709
	Chemotherapy TEC	5 (12.8)	34 (87.2)	
	Total number	8 (10.3)	70 (89.7)	

Abbreviations: CTC, circulating tumor cell; NCT, neoadjuvant chemotherapy; EC‐>T: four cycles of epirubicin (90 mg/m^2^)/cyclophosphamide (600 mg/m^2^) followed by four cycles of docetaxel (100mg/m^2^); TEC: six cycles of epirubicin (75 mg/m^2^)/cyclophosphamide(600 mg/m^2^)/docetaxel (75 mg/m^2^).

**Table 4:** The CTC status among four molecular subtypes pre‐ and post‐NCT

	CTC+/CTC+	CTC+/CTC‐	CTC‐/CTC+	CTC‐/CTC‐	*P*
ER/PR + HER2‐	0	1	0	14	.043
ER/PR + HER2+	2	5	1	17	
ER/PR‐ HER2+	3	8	0	11	
TNBC	3	7	0	6	

Abbreviations: CTC, circulating tumor cell; CTC−/CTC+, CTC‐negative both pre‐ and postchemotherapy; CTC−/CTC+, negative CTC converted to positive after chemotherapy; CTC+/CTC−, positive CTC converted to negative after chemotherapy; CTC+/CTC+, CTC‐positive both pre‐ and postchemotherapy; ER, estrogen receptor status; HER2, human epidermal growth factor receptor 2; NCT, neoadjuvant chemotherapy; PR, progesterone receptor status; TNBC, triple‐negative breast cancer.

### 3.3 Association between CTC dynamics and chemotherapy response rate

Afterwards, we evaluated the consistency between CTC dynamics pre‐ and postchemotherapy and the chemotherapy response rate (Table 5). Seven patients were CTC‐positive at baseline and before surgery, and imaging evaluation with MRI found that three patients had a PR, two patients had a CR, and two patients had SD. Interestingly, one patient reached a pathological complete response (pCR) in this group; one patient was CTC‐negative initially but found 3 CTCs/7.5 mL PB after NCT, and imaging was evaluated as SD before surgery. In addition, 22 CTC‐positive patients were found to be CTC‐negative after NCT, while nine patients reached PR (40.9%), 12 patients reached CR (54.5%), and one patient was evaluated as SD. The pathological assessment proved pCR in eight cases (36.4%). On the other hand, 48 patients were CTC‐negative both pre‐ and postchemotherapy, while 32 patients reached PR (66.7%), 11 patients reached CR (22.9%), and five patients were evaluated as SD (10.4%). The pathological assessment proved that nine cases reached pCR (18.8%). Meanwhile, the ORR of each group is also presented in Table 6. Herein, our data revealed a significant higher ORR in patients with CTC‐negative conversion after NCT (*P* = .013, Table 5), and the pCR rate revealed a borderline statistically significant higher in patients with positive CTC converted to negative after chemotherapy (*P* = .0608, Table 6).

**Table 5:** The relationship between CTC dynamics and chemotherapy response rate during neoadjuvant chemotherapy

The changes of CTC before and after NCT	n	PR (%)	CR (%)	ORR^a^ (%)	SD(%）	χ^2^	*P*
Positive/positive	7	3 (42.8)	2 (28.6)	5 (71.4)	2 (28.6)	10.773^b^	.013^b^
Positive/negative	22	9 (40.9)	12 (54.5)	21 (95.5)	1 (4.5)		
Negative/positive	1	0	0	0 (0)	1 (100)		
Negative/negative	48	32 (66.7)	11 (22.9)	43 (89.6)	5 (10.4)		

Abbreviations: CR, complete response; CTC, circulating tumor cell; n, number; NCT, neoadjuvant chemotherapy; ORR, partial response and complete response; PR, partial response; SD, stable disease.

a ORR = PR + CR.

b ORR vs SD among four groups.

**Table 6:** The relationship between CTC and the pCR rate

The changes of CTC before and after NCT	n	Non‐pCR（%）	pCR（%）	χ^2^	*P*
Positive/positive	7	7 (100)	0	3.515	.0608^a^
Positive/negative	22	14 (63.6)	8 (36.4)		
Negative/positive	1	1 (100)	0		
Negative/negative	48	39 (81.2)	9 (18.8)		

Abbreviations: CTC, circulating tumor cell; n: number; NCT, neoadjuvant chemotherapy; pCR, pathological complete response.

a CTC‐positive/negative vs CTC‐positive/positive group.

### 3.4 CTC detection and DFS

**Figure 1:**
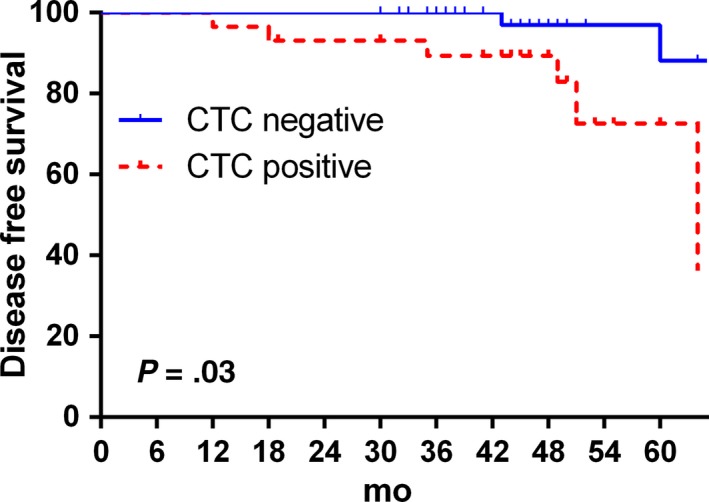
Kaplan‐Meier plots for disease‐free survival according to baseline circulating tumor cell

**Figure 2:**
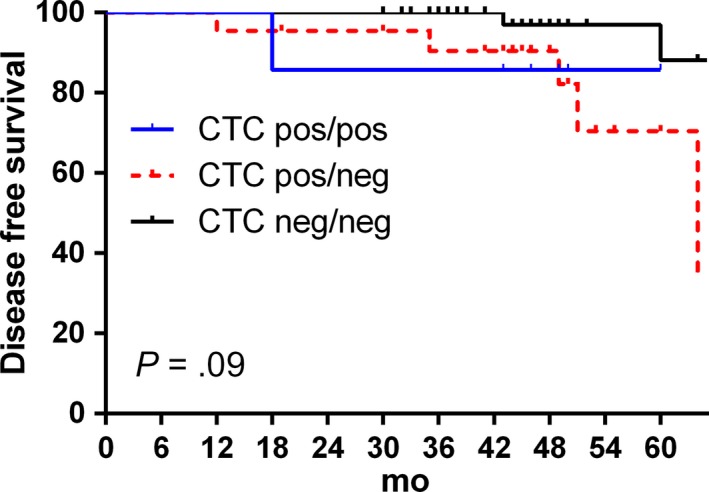
Kaplan‐Meier plots for disease‐free survival according to circulating tumor cell change pre‐ and postchemotherapy

The DFS data were also analyzed here. The average follow‐up time was 46.3 months (30‐64 months). We found that the prognosis was much worse in patients with positive CTC pretreatment (*P* = .003); there were six relapse events in patients with positive CTC and only two relapse events in patients with negative CTC (*P* = .03, Figure 1). However, our data did not reveal a significant relationship between DFS and CTC changes pre‐ and postchemotherapy (*P* = .09, Figure 2).

## 4 DISCUSSION

The prognostic value of CTCs has been proven in abundant studies, especially in patients with advanced BC.^2^, ^28^, ^29^ However, there is still a lack of consistent conclusions regarding whether CTCs could be applied as an independent predictor of NCT effectiveness in early BC. In this study, we used the CanPatrol technique to determine the relationship between NCT and CTCs in early BC. Our research found that a positive CTC result was related to patients' age, tumor size, TNM staging, and molecular classification at the time of diagnosis. In addition, the EC‐>T and TEC regimens have similar negative conversion ratios of CTCs, and most importantly, based on CTC dynamic evaluation, we found that the rate of ORR and pCR was much higher in patients with positive CTCs that converted to negative CTCs after NCT. Our data revealed that dynamic evaluation of CTCs may be related to the efficiency of NCT.

Various cancer cells are characterized by early dissemination to distant organs, including colorectal cancer, hepatocellular carcinoma, and BC.^30^, ^31^, ^32^ Hematogenous dissemination is one of the most important distant metastases based on the circulation*.* 33 CTC assessments have been applied in multiple steps of tumor treatment throughout early relapse, therapeutic efficacy, resistance occurrence, and selection of targeted drugs.^34^ Being regarded as the pre‐stadium to initiate metastases in distant organs,^12^ CTC populations in the blood of carcinoma patients contain cells with a clonal capacity.^35^, ^36^ Enumeration of CTCs during BC treatment has been used as a biomarker to reflect effective doses of antitumor agents,^37^ and CTCs may also serve as a monitor of drug susceptibility as tumors acquire mutations accompanying treatment in genes such as PIK3CA and estrogen receptor gene (ESR1).^38^ Therefore, our research evaluated disease progression and chemotherapeutic efficacy in early BC based on CTC detection.

Although a variety of CTC detection methods have been explored to increase the sensitivity and specificity of CTC detection, such as immunocytochemistry (ICC) and reverse transcription polymerase chain reaction (RT‐PCR),^24^ CellSearch is currently the most popular system for CTC enumeration.^39^ CTCs have been found to be composed of heterogeneous populations, including epithelial type, mesenchymal type, and intermediate state cells, and mesenchymal type was reported to possess cancer stem cell‐like properties,^40^ thus suggesting that they are more correlated with disease progression.^41^ Moreover, circulating tumor mammospheres (CTMs) are tumor cell clusters in the circulation and are associated with greater metastatic potential.^42^ However, only CTCs expressing epithelial cell adhesion molecule (EpCAM) will be identified with the CellSearch system.^39^, ^43^ Compared with the CellSearch platform, which uses anti‐EpCAM‐coated magnetic beads to capture CTCs, one study found that patients with an increased percentage of mesenchymal CTCs (M‐CTCs) after treatment had a worse prognosis based on CanPatrol technology.^44^ The CanPatrol technique could identify CTCs expressing both epithelial‐ and mesenchymal type‐related antigens. Moreover, as a result of 99.98% of leukocytes being depleted and a low number of leukocytes remaining on the membrane, it is easier to observe CTCs under a microscope with the CanPatrol technique.^45^ Therefore, our research applied the CanPatrol system to monitor the dynamic changes in CTC counts during NCT, which may yield a more precise result.

NCT is given to downstage the tumor for better locoregional control and increase the conservative surgery rate in early BC patients,^46^, ^47^, ^48^ but controversial concerns exist about the impact of NCT on CTCs in BC. A cohort of 27 patients with early stage BC underwent NCT for three courses, and the CTCs were evaluated after each chemotherapy cycle. The results showed an increasing tendency regardless of the total CTC number, stem cell‐like (CD44+ CD24−), or EMT‐type CTCs (N‐cadherin+).^40^ In addition, an in vivo animal experiment also indicated that NCT could promote luminal BC cell intrusion into the circulation.^49^ On the other hand, a meta‐analysis included 1574 BC patients who underwent NCT and evaluated the CTC numbers with CellSearch at baseline and before surgery; although there was no significant correlation between the pCR and CTC detection before surgery, the CTC count was lower than the baseline count (*P* < .001).^10^ Here, our results also revealed that CTC numbers decreased with NCT, and we did not find a definite correlation between CTC changes and the pCR rate, but the patients with positive CTCs at baseline and converted to negative CTCs after NCT had a higher pCR ratio compared to that in patients in the other groups, which suggested a potential pCR predictive value of CTC numeration in patients with CTCs detected before NCT. Furthermore, although we failed to find a significant association between DFS and CTC dynamic changes, all eight patients achieved both pCR and CTC‐negative conversion after NCT and were recurrence‐free within the follow‐up period. The BEVERLY‐2 trial^50^ was a prospective study that included 52 inflammatory BC patients, which is a very aggressive subtype of BC, and found that CTC‐positivity (>1 CTC/7.5 mL) dropped from 35% to 7% with NCT. Patients with at least one positive CTC had a DFS of 54%, compared with a DFS of 83% in patients where no CTCs were detected during neoadjuvant stages (*P* = .018). These results indicated a favorable prognosis in this subpopulation. However, regardless of the BEVERLY‐2 or Geparquattro trial, the CTCs were evaluated with the CellSearch system, which is only able to detect CTCs with EpCAM and keratin expression. CTCs that have completely lost their epithelial features will not be identified with the CellSearch system. Therefore, our results offer suitable evidence of the value of CTC monitoring in the NCT process, but definite conclusions require future prospective trials including larger cohorts.

## 5 CONCLUSION

In conclusion, this study revealed that CTCs are associated with tumor size, tumor stage, and molecular classification at the time of diagnosis. Although the study sample in our research is relatively small, it preliminarily provides evidence of the relevance between CTC status and the overall response rate of NCT in early BC.

## CONFLICT OF INTEREST

The authors declare that there are no conflict of interest.
