# Supplementary material for: Prospective study of the relevance of circulating tumor cell status and neoadjuvant chemotherapy effectiveness in early breast cancer
Source: Cancer Med. 2020 Feb 4;9(7):2290–8. doi: 10.1002/cam4.2876 (PMC7131845; doi:10.1002/cam4.2876)
Supplement: Supplementary file 1 [file CAM4-9-2290-s001.docx]

**TableS1. The phenotype of CTCs before and after neoadjuvant chemotherapy**

| No. | Molecular subtype | Base line CTC numbers | | | | Preoperative CTC numbers | | | | P value |
| --- | --- | --- | --- | --- | --- | --- | --- | --- | --- | --- |
|  |  | E+ | B+ | M+ | Total | E+ | B+ | M+ | Total | <0.001 |
| 1 | ER/PR+ HER2+ | 2 | 0 | 0 | 2 | 1 | 0 | 0 | 1 |  |
| 2 | ER/PR+ HER2- | 1 | 1 | 1 | 3 | 0 | 0 | 0 | 0 |  |
| 3 | ER/PR- HER2+ | 0 | 1 | 2 | 3 | 0 | 0 | 0 | 0 |  |
| 4 | TNBC | 0 | 1 | 0 | 1 | 0 | 0 | 0 | 0 |  |
| 5 | ER/PR+ HER2+ | 3 | 0 | 2 | 5 | 0 | 2 | 0 | 2 |  |
| 6 | ER/PR+ HER2+ | 2 | 0 | 1 | 3 | 0 | 0 | 0 | 0 |  |
| 7 | ER/PR- HER2+ | 1 | 1 | 0 | 2 | 1 | 0 | 0 | 1 |  |
| 8 | TNBC | 2 | 2 | 3 | 7 | 0 | 0 | 1 | 1 |  |
| 9 | ER/PR- HER2+ | 1 | 3 | 2 | 6 | 0 | 0 | 0 | 0 |  |
| 10 | ER/PR+ HER2+ | 0 | 2 | 0 | 2 | 0 | 0 | 0 | 0 |  |
| 11 | ER/PR- HER2+ | 1 | 3 | 1 | 5 | 0 | 0 | 0 | 0 |  |
| 12 | TNBC | 0 | 2 | 1 | 3 | 0 | 0 | 0 | 0 |  |
| 13 | ER/PR- HER2+ | 1 | 1 | 2 | 4 | 1 | 1 | 0 | 2 |  |
| 14 | TNBC | 1 | 1 | 1 | 3 | 0 | 0 | 0 | 0 |  |
| 15 | TNBC | 4 | 0 | 0 | 4 | 1 | 0 | 0 | 1 |  |
| 16 | ER/PR+ HER2+ | 0 | 2 | 2 | 4 | 0 | 0 | 0 | 0 |  |
| 17 | ER/PR- HER2+ | 2 | 0 | 1 | 3 | 0 | 0 | 0 | 0 |  |
| 18 | ER/PR+ HER2+ | 1 | 0 | 0 | 1 | 0 | 0 | 0 | 0 |  |
| 19 | TNBC | 3 | 1 | 0 | 4 | 0 | 0 | 0 | 0 |  |
| 20 | ER/PR+ HER2+ | 0 | 0 | 0 | 0 | 2 | 0 | 1 | 3 |  |
| 21 | TNBC | 0 | 0 | 2 | 2 | 0 | 0 | 0 | 0 |  |
| 22 | ER/PR- HER2+ | 5 | 0 | 0 | 5 | 0 | 0 | 0 | 0 |  |
| 23 | ER/PR- HER2+ | 1 | 0 | 3 | 4 | 0 | 1 | 1 | 2 |  |
| 24 | TNBC | 1 | 0 | 0 | 1 | 0 | 0 | 0 | 0 |  |
| 25 | ER/PR- HER2+ | 2 | 0 | 0 | 2 | 0 | 0 | 0 | 0 |  |
| 26 | ER/PR- HER2+ | 2 | 2 | 1 | 5 | 2 | 0 | 0 | 2 |  |
| 27 | TNBC | 0 | 2 | 1 | 3 | 0 | 0 | 0 | 0 |  |
| 28 | ER/PR+ HER2+ | 1 | 1 | 2 | 4 | 0 | 0 | 0 | 0 |  |
| 29 | TNBC | 3 | 0 | 1 | 4 | 3 | 0 | 0 | 3 |  |
| 30 | ER/PR- HER2+ | 1 | 0 | 1 | 2 | 0 | 0 | 0 | 0 |  |
| Total |  | 41 | 26 | 30 | 97 | 11 | 4 | 3 | 18 |  |

**Abbreviation** CTC: circulating tumor cell; ER: estrogen receptor status; PR: progesterone receptor status; HER2: human epidermal growth factor receptor 2; TNBC: triple negative breast cancer; E+, epithelial type; M+, mesenchymal type; B+, biophenotypic type; There are totally 30 patients listed here, including one patient who had zero CTC in the beginning and positive CTCs after NCT
